# Risk of Hypophosphatemia Following Postpartum Anemia Treatment with IV Ferric Carboxymaltose, IV Ferric Derisomaltose, and Oral Ferrous Sulfate

**DOI:** 10.3390/jcm14238393

**Published:** 2025-11-26

**Authors:** Lea Bombač Tavčar, Miha Lučovnik, Hana Hrobat, Lea Gornik, Irena Preložnik Zupan

**Affiliations:** 1 Department of Perinatology, Division of Gynecology and Obstetrics, University Medical Centre Ljubljana, Šlajmerjeva 3, 1000 Ljubljana, Slovenia; miha.lucovnik@kclj.si; 2 Faculty of Medicine, University of Ljubljana, Vrazov trg 2, 1000 Ljubljana, Slovenia; hana.hrobat@icloud.com (H.H.); lea.gornik@gmail.com (L.G.); irena.zupan@kclj.si (I.P.Z.); 3 Department of Hematology, University Medical Centre Ljubljana, Zaloška 7, 1000 Ljubljana, Slovenia

**Keywords:** hypophosphatemia, postpartum anemia, IV ferric derisomaltose, IV ferric carboxymaltose, oral ferrous sulfate

## Abstract

**Objective**: To compare the effects of intravenous (IV) ferric carboxymaltose (FCM), IV ferric derisomaltose (FDI), and oral ferrous sulfate on hypophosphatemia in women with postpartum anemia. **Methods**: Single-centre, open-label, randomized trial. Women were randomly allocated to receive IV FCM, IV FDI, or oral ferrous sulfate. IV iron was infused in one or two doses (on days 0 and 7 postpartum), while ferrous sulfate once daily. Serum phosphate level was measured at enrollment and 6 weeks postpartum. Serum phosphate level and the proportion of women with hypophosphatemia were analyzed. The Kruskal–Wallis test was used for comparison of phosphate levels and the Chi-squared test for comparison of proportions (*p* < 0.05). **Results**: Three-hundred women with postpartum anemia (Hb < 100 g/L within 48 h postpartum) were included. Phosphate levels at six weeks postpartum did not differ between groups: 1.24 mmol/L (inter-quartile range (IQR) 1.10–1.35; equivalent to 3.84 mg/dL, IQR 3.41–4.19) in the FCM group, 1.23 mmol/L (IQR 1.09–1.35; equivalent to 3.81 mg/dL, IQR 3.38–4.19) in the FDI group, and 1.25 mmol/L (IQR 1.15–1.34; equivalent to 3.88 mg/dL, IQR 3.57–4.15) in the ferrous sulfate group (*p* = 0.86). The proportion of women with hypophosphatemia was similarly low in all three groups (3 (3.1%) vs. 2 (2.2%) vs. 2 (2.2%); *p* = 0.89). At six weeks postpartum, hemoglobin levels were slightly higher in both IV iron groups compared with oral iron, but the differences were small and unlikely to be clinically meaningful. **Conclusions**: Treatment of postpartum anemia with IV FCM, IV FDI, and oral ferrous sulfate had similar impact on phosphate levels and incidence of hypophosphatemia at six weeks postpartum.

## 1. Introduction

Postpartum iron deficiency and iron deficiency anemia (IDA) are frequent maternal complications, affecting up to 50% of women in developed countries and potentially reaching 80% in developing regions [1,2,3]. Effective iron supplementation represents an essential component of high-quality postpartum care. Oral iron remains the standard first-line treatment option for stable, asymptomatic, or mildly symptomatic postpartum anemia, typically administered at a dose between 40 and 200 mg of elemental iron daily for three months [4]. Intravenous (IV) iron therapy should be considered in cases of oral iron intolerance, poor adherence, malabsorption (e.g., due to inflammation, chronic kidney disease, bariatric surgery, or other conditions impairing intestinal iron uptake), or when rapid correction of iron deficiency is needed. [5]. Blood transfusions are reserved for women who develop hemodynamic instability due to postpartum hemorrhage [4,6,7].

High-dose parenteral iron administration allows rapid and efficient replenishment of iron stores and can reduce the need for postpartum red blood cell transfusion [4]. In recent years, modern IV iron formulations have been approved for clinical use to rapidly correct IDA, including ferric carboxymaltose (FCM) and ferric derisomaltose (FDI) [3,8]. These agents can be administered in single doses of up to 1 g for FCM and 1.5 g for FDI within approximately 15 min, with a low incidence (<1%) of immediate infusion-related reactions [9,10]. This represents a major improvement over older formulations such as iron sucrose, which is limited to 200 mg per infusion, and iron dextran, which can be given at higher doses but requires prolonged administration [11]. Several studies have demonstrated the superiority of these modern IV iron formulations over oral iron, as iron stores can be restored with one or two infusions, whereas oral therapy typically requires several months and is frequently associated with gastrointestinal adverse effects [5,12,13,14,15,16,17,18].

Hypophosphatemia has been documented as a potential adverse event of novel IV iron treatments affecting 50–74% of patients treated with FCM in prospective clinical trials [9]. Hypophosphatemia is defined as a serum phosphate level less than 0.83 mmol/L, with values <0.34 mmol/L considered severe [5,18,19,20]. This condition is mediated by increased secretion of the phosphaturic hormone fibroblast growth factor 23 (FGF-23), which initiates a cascade leading to renal phosphate wasting [21,22,23,24]. The so-called “6H syndrome” encompasses hyperphosphaturic hypophosphatemia driven by elevated FGF-23 levels, resulting in hypovitaminosis D, hypocalcemia, and secondary hyperparathyroidism. These disturbances may lead to potentially irreversible clinical consequences, including bone pain, osteomalacia, and fractures [9].

Risks of hypophosphatemia in postpartum women following IV FMC, IV FDI, and oral ferrous sulfate treatments have not yet been compared head-to-head. Our randomized controlled trial sought to assess and compare the impact of these three iron formulations used to treat postpartum IDA on phosphate levels measured six weeks postpartum.

## 2. Materials and Methods

### 2.1. Study Design

This was a prospective, single-centre, open-label randomized trial conducted at the Department of Perinatology, University Medical Centre Ljubljana, Slovenia. Participant enrollment and follow-up were carried out between September 2020 and March 2022. The study protocol received approval from the National Medical Ethics Committee (approval number: 0120-117/2019/5; 17 April 2019). Written informed consent was obtained from all participants prior to inclusion. The study was conducted in accordance with the Declaration of Helsinki and was registered at ClinicalTrials.gov (identifier: NCT03957057).

### 2.2. Participants

We recruited postpartum women aged 18 to 46 years with iron deficiency anemia (IDA), defined by a hemoglobin (Hb) concentration between 70 and 100 g/L measured within 48 h after childbirth. Women were excluded if they had a documented cause of anemia other than iron deficiency, postpartum hemorrhage exceeding 500 mL, signs of acute infection, known hypersensitivity to the administered iron formulations, or clinically relevant renal or hepatic impairment.

### 2.3. Randomization and Blinding

Participants were randomly assigned to one of three treatment arms in a 1:1:1 ratio. Allocation was performed using sequentially numbered, opaque envelopes prepared according to a computer-generated randomization sequence. IV medications were prepared and administered exclusively by nursing staff not otherwise involved in the study procedures. Due to the nature of the interventions, blinding of participants and investigators was not feasible. Participants received one of the following treatments: IV FCM (Iroprem^®^, Sandoz, Ljubljana, Slovenia), IV FDI (Monofer^®^, Ewopharma, Zürich, Switzerland), or oral ferrous sulfate (Tardyferon^®^, Pierre Fabre Médicament, Lavaur, France). For women assigned to IV therapy, the total iron requirement was calculated using a modified Ganzoni formula [25]—pre-pregnancy weight (kg) × (15 − baseline Hb) × 2.4 + 500 mg—to correct anemia and replenish iron stores while minimizing the risk of overdosing. The calculated dose of IV iron administered was rounded to the nearest 500 mg increment (1000, 1500, or 2000 mg). In accordance with manufacturer recommendations, the maximal allowable dose administered in a single day was 1000 mg for FCM and 1500 mg for FDI. If the total calculated requirement exceeded these limits, the remaining dose was administered one week later. Both IV formulations were diluted in 250 mL of isotonic saline and infused over approximately 15–30 min. Women randomized to the oral iron group were instructed to take 160 mg of ferrous sulfate once daily (two 80 mg tablets), preferably with a source of vitamin C and at least one hour before meals, from study inclusion until six weeks postpartum.

### 2.4. Study Outcomes

The primary outcome of the study was the proportion of participants with hypophosphatemia, defined as a serum phosphate concentration < 0.83 mmol/L (equivalent to <2.57 mg/dL) six weeks postpartum.

The pre-specified secondary outcomes were:

- Serum phosphate level at six weeks postpartum.
- The proportion of participants with severe hypophosphatemia, defined as <0.34 mmol/L (equivalent to <1.05 mg/dL), at six weeks follow-up.
- The change in serum phosphate level from study inclusion to six weeks postpartum.

In addition, adverse events related to study treatments were recorded, and adherence to the prescribed oral iron regimen was evaluated.

### 2.5. Sample Size

The sample size calculation was based on previously reported rates of hypophosphatemia, estimated at approximately 15% for FDI and 40% for FCM [23]. To detect a statistically significant difference between the treatment arms with 80% power and α of 5%, a minimum of 98 participants per group was required. To account for potential loss to follow-up, the planned enrollment target was set at 100 women per treatment group.

### 2.6. Statistical Analysis

All data were collected according to a pre-specified statistical plan and followed the intention-to-treat principle. Participants who required blood transfusion for symptomatic anemia after enrollment, as well as those who did not attend the six-week postpartum follow-up, were excluded from the final dataset. Continuous variables were compared using the Kruskal–Wallis test, with *Tamhane’s* T2 test used for post hoc pairwise comparisons. Categorical variables were analyzed using the Chi-square test. A *p* < 0.05 was considered statistically significant. All analyses were performed using SPSS software (version 28.0; IBM Corporation, Armonk, New York, NY, USA).

## 3. Results

**Figure 1:**
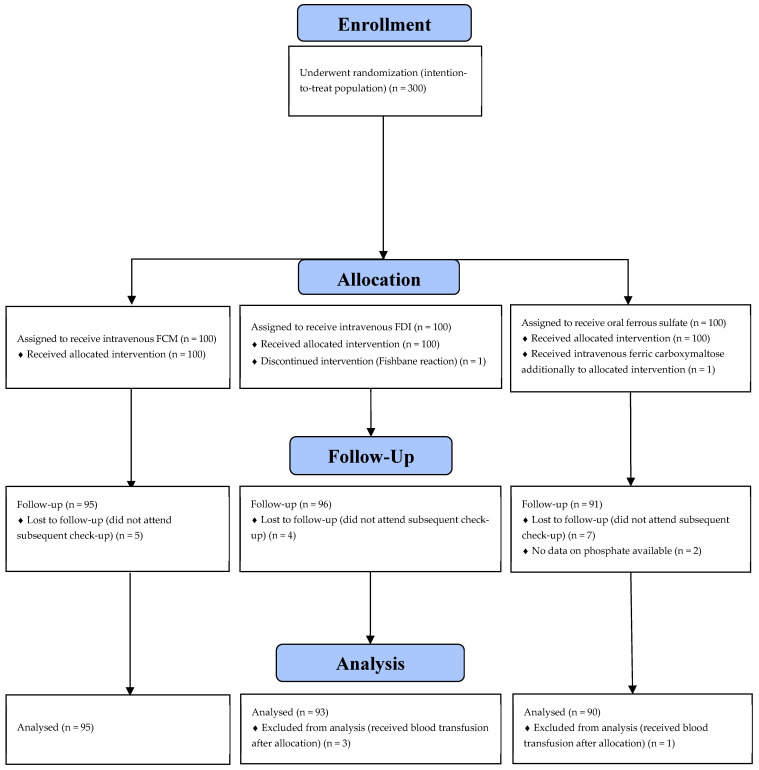
CONSORT diagram for study participation. FCM—ferric carboxymaltose; FDI—ferric derisomaltose.

A total of 300 women were enrolled and randomized. Primary outcome data were unavailable for 18 participants: 16 did not attend the six-week postpartum follow-up visit, and serum phosphate levels were not measured at the six-week assessment in two participants. In addition, four participants were excluded from the final analysis for receiving blood transfusions after study inclusion (Figure 1). Eventually, 278 of 300 enrollees (93%) completed the trial. All women included in the study were Caucasian.

Demographic and clinical characteristics were generally comparable among the three treatment groups. The only notable difference was a statistically higher baseline Hb concentration in the ferrous sulfate group (Table 1). Median calculated iron deficit at enrolment was similar in both IV groups (1422 g in the FCM group and 1437 g in the FDI group, *p* = 0.26). In the FCM group, 74.7% (71 out of 95) participants received two doses of FCM. The rest needed only one dose of FCM. All participants received 1000 mg of FCM as the first dose. Of those who needed a second dose, 8.4% (6 out of 71) received 1000 mg of FCM. In the FDI group, only 6.4% (6 out of 93) of participants needed an additional application of FDI. Baseline phosphate levels and proportions of participants with hypophosphatemia at inclusion were similar in the three groups (Table 1). Eight participants had a phosphate level of <0.83 mmol/L (equivalent to 2.57 mg/dL) at inclusion, with similar proportions among groups (Table 1).

Transfusion rates prior to study inclusion were comparable across all treatment groups. Overall, 18 of 278 participants (6.5%) required transfusion before enrollment (Table 1).

Table 2 presents a comparison of phosphate levels between study groups six weeks after treatment. Hypophosphatemia was reported in seven patients, at similar low frequencies among groups: 3.1% (3 out of 95) in the FCM group, vs. 2.2% (2 out of 93) in the FDI group, vs. 2.2% (2 out of 90) in the oral iron group. No participant in the IV groups received more than 15 mg/kg of FCM or 20 mg/kg of FDI.

In addition to phosphate measurements, hemoglobin concentrations at six weeks postpartum were assessed across all treatment groups. Median hemoglobin levels (IQR) were 135 g/L (131–139) in the FCM group, 134 g/L (128–139) in the FDI group, and 131 g/L (125–137) in the oral ferrous sulfate group. A significant difference was observed between groups (Kruskal–Wallis test, *p* = 0.002); however, post hoc pairwise comparisons (Tamhane T2) showed no difference between FCM and FDI (*p* = 0.60), while both IV iron formulations resulted in slightly higher hemoglobin levels compared with oral iron (FCM vs. ferrous sulfate: *p* = 0.0003; FDI vs. ferrous sulfate: *p* = 0.003). Although statistically significant, these differences were small and are unlikely to be clinically meaningful.

## 4. Discussion

Identification and optimal management of postpartum anemia and iron deficiency are crucial for optimal recovery of the parturient. We studied the occurrence of hypophosphatemia in 300 anemic postpartum women six weeks after starting the treatment with one of three different iron formulations: IV FCM, IV FDI, or oral ferrous sulfate. To the best of our knowledge, this is the first randomized study of postpartum anemia treatment with a head-to-head comparison of IV FCM and FDI in terms of their effects on serum phosphate levels in women after childbirth. Although there are numerous case reports of skeletal complications with IV iron formulations [19,20,26] and bone turnover biomarkers [23], no previous controlled studies examined the effects of IV iron on the incidence of hypophosphatemia in a postpartum population. We found no significant difference in phosphate levels following treatments among the three study groups. Furthermore, in contrast to studies in other populations [18,22,23,27], there was no difference in the proportion of participants experiencing hypophosphatemia six weeks postpartum among the study groups. In total, only seven women (2.2–3.1% of participants in each group) developed hypophosphatemia. Moreover, all eight participants with low phosphate levels at inclusion (<0.83 mmol/L) (<2.57 mg/dL) had normal phosphate levels at six weeks postpartum, regardless of treatment received. On the other hand, all participants with low phosphate levels after therapy (<0.83 mmol/L) (<2.57 mg/dL) had a phosphate level of >1 mmol/L (>3.1 mg/dL) at study inclusion. Severe hypophosphatemia six weeks postpartum (phosphate level 0.29 mmol/L) (0.90 mg/dL) was observed in just one 41-year-old participant included in the oral ferrous sulfate group, who had normal phosphate levels before treatment (1.43 mmol/L) (4.43 mg/dL). She had no comorbidities but was moderately anemic after childbirth, with a hemoglobin concentration of 92 g/L at enrollment and hemoglobin 143 g/L six weeks postpartum.

Our study is consistent with large field trials conducted in Malawi among pregnant women with anemia, which compared intravenous ferric carboxymaltose (FCM) with oral iron supplementation. These studies showed that hypophosphatemia following FCM administration was uncommon, transient, and clinically insignificant. In a randomized controlled trial during the second trimester, the prevalence of moderate hypophosphatemia four weeks after FCM was slightly higher than with standard care (5% vs. 2%), but this difference resolved at later timepoints [28]. Similarly, a subsequent trial in the third trimester confirmed the safety of FCM, reporting no clinically meaningful differences in phosphate levels or serious adverse events [29]. These findings further support that in pregnant or recently postpartum women, physiological adaptations may attenuate the phosphate-lowering effects of FCM observed in non-pregnant adults. Our findings contrast with previous studies comparing clinical outcomes in some patient populations, e.g., patients with inflammatory bowel disease [30] or chronic kidney disease [31] treated with FCM vs. FDI. These trials reported that FCM may lead to a reduction in serum phosphate levels. On the other hand, our results agree with several other studies. Van Wyck et al. [16] compared FCM and oral iron for postpartum anemia in 361 patients. They noted a transient fall in serum phosphate levels among patients in the FCM treatment group. The nadir was reached at day 14 after treatment, with a return to normal phosphate levels by day 42. Hypophosphatemia after IV FCM administration for postpartum iron deficiency anemia was asymptomatic and reversed spontaneously without specific treatment. In their multicenter, randomized trial, Seid et al. (2008) [14] investigated efficacy, safety, and tolerability of IV FCM compared with oral iron in nearly 300 women with postpartum anemia. A transient, asymptomatic lowering of serum phosphate was observed in both treatment groups, although significantly greater in the FCM group, with a nadir reached two weeks after initiating therapy and spontaneously resolving thereafter. In the subsequent multicenter study by Seid et al. (2017) [13], the authors compared IV FCM treatment to standard of care involving oral iron formulations for women with postpartum anemia and women with heavy menstrual bleeding and anemia. Hypophosphatemia was identified in 6 out of 996 participants in the FCM group, all six in the heavy menstrual bleeding group.

Absence of difference in maternal phosphate levels in the three treatment groups observed in our study could be explained by numerous factors that impact serum phosphate levels. Postpartum women are young and mostly healthy with normal renal function and do not require repeated iron infusions, which is one of the risk factors for prolonged hypophosphatemia and resulting osteomalacia [18,20,22,23,24]. Postpartum IDA is caused by low storage of iron due to pregnancy and postpartum hemorrhage, which is easily replenished by one or two infusions of IV iron formulations. Another factor that could explain our results is the relatively high serum phosphate levels at baseline. More than 90% of women had a phosphate level of >1 mmol/L (equivalent to >3.1 mg/dL) at inclusion. A high phosphate level is considered one of the most important safety elements in the decision whether to apply FCM or not, as it reduces the risk of hypophosphatemia related to FCM treatment [18,27]. Hormonal changes during pregnancy and postpartum could also have a protective effect against hypophosphatemia. Indeed, there are some data suggesting that breastfeeding may cause an increase in serum phosphate levels because of increased reabsorption of phosphate by the kidney and accelerated resorption from the skeleton [32].

Our study has several limitations. We only measured phosphate levels six weeks after treatments. Therefore, a higher proportion of participants may have developed transient hypophosphatemia, which resolved spontaneously by the six-week check-up visit. Studies have indicated that nadir serum phosphate in patients given IV FCM occurs approximately 2 to 3 weeks after administration. However, research has also shown that a significant percentage of patients continue to manifest severe hypophosphatemia at five weeks [22,33]. A potential limitation of our study is that serum phosphate levels were measured only at six weeks postpartum, which did not allow for monitoring the earlier dynamics of phosphate changes. Nevertheless, even if transient hypophosphatemia had occurred earlier and resolved by six weeks, such episodes appear to be clinically insignificant, as they resolved spontaneously and were not associated with any complications. The six-week postpartum timepoint was deliberately chosen because, in our national clinical practice, all women attend a routine postpartum medical check-up at this stage. We therefore aimed to design a study that reflects real-world clinical practice and provides data of practical relevance for everyday obstetric care. The generalizability of our findings may be limited to otherwise healthy postpartum women. Results might differ in populations with renal impairment or other comorbidities affecting phosphate metabolism. Other limitations of the study should also be considered. We did not assess clinical outcomes of hypophosphatemia and did not follow the participants over a longer period. However, there were only a few cases of hypophosphatemia distributed equally among groups, and no cases of severe hypophosphatemia in the IV iron groups. Therefore, it is safe to assume that the risk of osteomalacia, fractures, and other clinical complications of hypophosphatemia occurs extremely rarely in postpartum women treated with IV iron medications. Another limitation of the study is the lack of a placebo control group. Nevertheless, given the large body of evidence on adverse health consequences of untreated postpartum anemia [1,10], the inclusion of a placebo arm was deemed unethical. Future studies including earlier and multiple timepoints for phosphate assessment, as well as populations with comorbidities affecting phosphate metabolism, may provide further insights into the temporal and clinical aspects of post-treatment phosphate changes.

## 5. Conclusions

In conclusion, phosphate levels at six weeks postpartum in our randomized study were not impacted by the type of iron medication used to treat postpartum IDA. The overall frequency of hypophosphatemia was very low, and no clinically significant or persistent cases were observed in women treated with intravenous iron. These results suggest that intravenous iron therapy can be considered safe and effective for the management of postpartum anemia in otherwise healthy women. Therefore, if IV FCM is considered for the correction of iron deficiency in women after childbirth, it seems that serum phosphate concentrations after treatment do not need to be measured routinely in this population.

## Figures and Tables

**Table 1:** Participant demographic and baseline characteristics (at study inclusion, before treatment).

Statistics/Category	Treatment Group			*p* Value
	IV FCM (n = 95)	IV FDI (n = 93)	Oral Ferrous Sulfate (n = 90)	
Maternal age, years	31 (27–35)	30 (27–34)	31 (27–36)	0.40
Prepregnancy BMI, kg/m^2^	24 (21–27)	24 (22–27)	23 (21–27)	0.63
BMI at delivery, kg/m^2^	28 (26–32)	30 (27–32)	29 (26–32)	0.54
Multiple gestation	6 (6)	6 (6)	3 (3)	0.57
Nulliparity	64 (67)	67 (72)	50 (56)	0.06
Gestational age at birth, completed weeks	39 (38–40)	39 (38–40)	39 (38–40)	0.66
Transfusion of RBC before intervention	5 (5)	6 (6)	7 (8)	0.79
Cesarean section	28 (29)	22 (24)	30 (33)	0.35
Calculated iron deficit ^1^, g	1422 (1280–1560)	1437 (1345–1582)	/	0.26
Hemoglobin, g/L	92 (87–97)	91 (87–95)	95 (91–98)	0.002
Ferritin, µ/L	28 (13–48)	21 (12–34)	22 (12–40)	0.17
Transferrin saturation, %	9 (7–14)	10 (7–15)	10 (7–16)	0.35
Plasma iron µmol/L	7 (4–10)	8 (5–12)	7 (5–11)	0.11
TIBC, µmol/L	71 (64–78)	73 (67–80)	71 (65–79)	0.31
CRP, mg/L	57 (35–95)	53 (33–78)	46 (33–71)	0.30
Baseline phosphate level, mmol/L or mg/dL	1.15 (1.01–1.33) 3.57 (3.13–4.12)	1.16 (1.04–1.30) 3.60 (3.22–4.03)	1.22 (1.08–1.35) 3.78 (3.35–4.19)	0.13
Hypophosphatemia at inclusion (<0.83 mmol/L) (equivalent to <2.57 mg/dL)	2 (2)	3 (3)	3 (4)	0.67
Severe hypophosphatemia at inclusion (<0.34 mmol/L) (equivalent to <1.05 mg/dL)	0 (0)	0 (0)	0 (0)	/

Data are presented as median (interquartile range) or n (%); FCM, ferric carboxymaltose; FDI, ferric derisomaltose; BMI, body mass index; RBC red blood cells; TIBC, total iron binding capacity; CRP, C-reactive protein; ^1^ Iron deficit calculated using Ganzoni formula.

**Table 2:** Comparison of phosphate levels between the three study groups (at six weeks following treatment).

Statistics/Category	Treatment Group			*p* Value
	IV FCM (n = 95)	IV FDI (n = 93)	Oral Ferrous Sulfate (n = 90)	
Hypophosphatemia (<0.83 mmol/L) (equivalent to 2.57 mg/dL) six weeks postpartum, %	3 (3)	2 (2)	2 (2)	0.89
Phosphate level six weeks postpartum, mmol/L or mg/dL	1.24 (1.10–1.35) 3.84 (3.41–4.19)	1.23 (1.09–1.35) 3.81 (3.38–4.19)	1.25 (1.15–1.34) 3.88 (3.57–4.15)	0.86
Severe hypophosphatemia (<0.34 mmol/L) (equivalent to <1.05 mg/dL) six weeks postpartum, %	0 (0)	0 (0)	1 (1)	0.35
Change in phosphate level from study inclusion to six weeks postpartum, mmol/L or mg/dL	−0.08 (−0.26–0.17) 0.25 (−0.81–0.53)	−0.09 (−0.26–0.08) −0.28 (−0.81–0.25)	−0.01 (−0.18–0.09) −0.03 (−0.56–0.28)	0.46

Data are presented as n (%) or median (interquartile range). FCM—ferric carboxymaltose; FDI—ferric derisomaltose.

## Data Availability

The datasets generated and analyzed during the current study are available from the corresponding author upon reasonable request in the preferred format. Data will be accessible from the time of publication for a minimum period of 15 years.

## Abbreviations

The following abbreviations are used in this manuscript:

IDA	iron deficiency anemia
IV	intravenous
FCM	ferric carboxymaltose
FDI	ferric derisomaltose
